# A Compromised Airway in the Setting of Failed Extracorporeal Membrane Oxygenation Cannulation

**DOI:** 10.7759/cureus.25164

**Published:** 2022-05-20

**Authors:** Matthew Edwards, Jason Gassman, John Rosasco, Patrick Kim, Aimee Pak

**Affiliations:** 1 Anesthesiology, University of Oklahoma Health Sciences Center, Oklahoma City, USA

**Keywords:** tracheal stenosis, difficult airway management, fiberoptic intubation, extracorporeal membrane oxygenation support, mediastinal tumors

## Abstract

We report a case involving failed extracorporeal membrane oxygenation (ECMO) cannulation in the setting of critical airway stenosis secondary to a large anterior mediastinal mass. The most invasive management option, ECMO, was initially pursued solely to avoid manipulation of a critical airway in case of intubation failure or life-threatening airway bleeding. However, after unexpectedly failing cannulation in two separate cannulation sites with an impending respiratory collapse, awake fiberoptic or emergent rigid bronchoscopy was the remaining viable option. The patient was ultimately intubated via awake fiberoptic intubation even though this modality carried a high complication risk and potential mortality if failed. This case report illustrates both the potential role of ECMO within the airway management hierarchy and the persistent need for contingency planning should ECMO cannulation fail. With the recent enthusiasm for ECMO incorporation into difficult airway management, our report serves to highlight the very serious issue of cannulation failure. There is a limited amount of case reports describing ECMO failure in a critical airway, and little has been described about rescue methods when ECMO fails. Our goal is to remind readers that although ECMO can be an excellent rescue option for a critically ill patient, it cannot be viewed as a last line of therapy. If one is able to rapidly recognize impending ECMO cannulation failure and is prepared for cannulation failure, they can save invaluable time in a decompensating patient.

## Introduction

When critical airway narrowing occurs below the vocal cords, oxygenation and ventilation may still be compromised if the tracheal tube is not advanced beyond the stenotic lumen. While the American Society of Anesthesiologists’ Difficult Airway Algorithm has traditionally been used for upper airway management [1], its guidance is limited for the distal airway. Extracorporeal membrane oxygenation (ECMO) has emerged as a therapy to provide cardiac or pulmonary support to patients with impaired perfusion or gas exchange, and several published reports have described successful ECMO implementation in cases of difficult airways or tracheal compromise [2-6]. Although there are many reports of successful ECMO cannulation prior to instrumenting difficult airways, there are limited examples of case reports describing when an ECMO attempt fails and is no longer a viable option. In this report, we present a case of a patient in acute respiratory failure with a difficult airway and critical airway stenosis secondary to an anterior mediastinal mass. Veno-venous ECMO under monitored anesthetic care was initially pursued for airway and respiratory control, but vascular cannulation was unable to be obtained. This was largely in part due to the patient’s body habitus, patient position, and possible anatomical variants. Other factors that have been cited that may have contributed to failure include low mean arterial pressures, hypovolemia, prior cannulation, and vascular calcifications or scarring [7]. Although cannulation failed, the patient was able to be intubated without complications using an awake fiberoptic nasal approach.

## Case presentation

**Figure 1:**
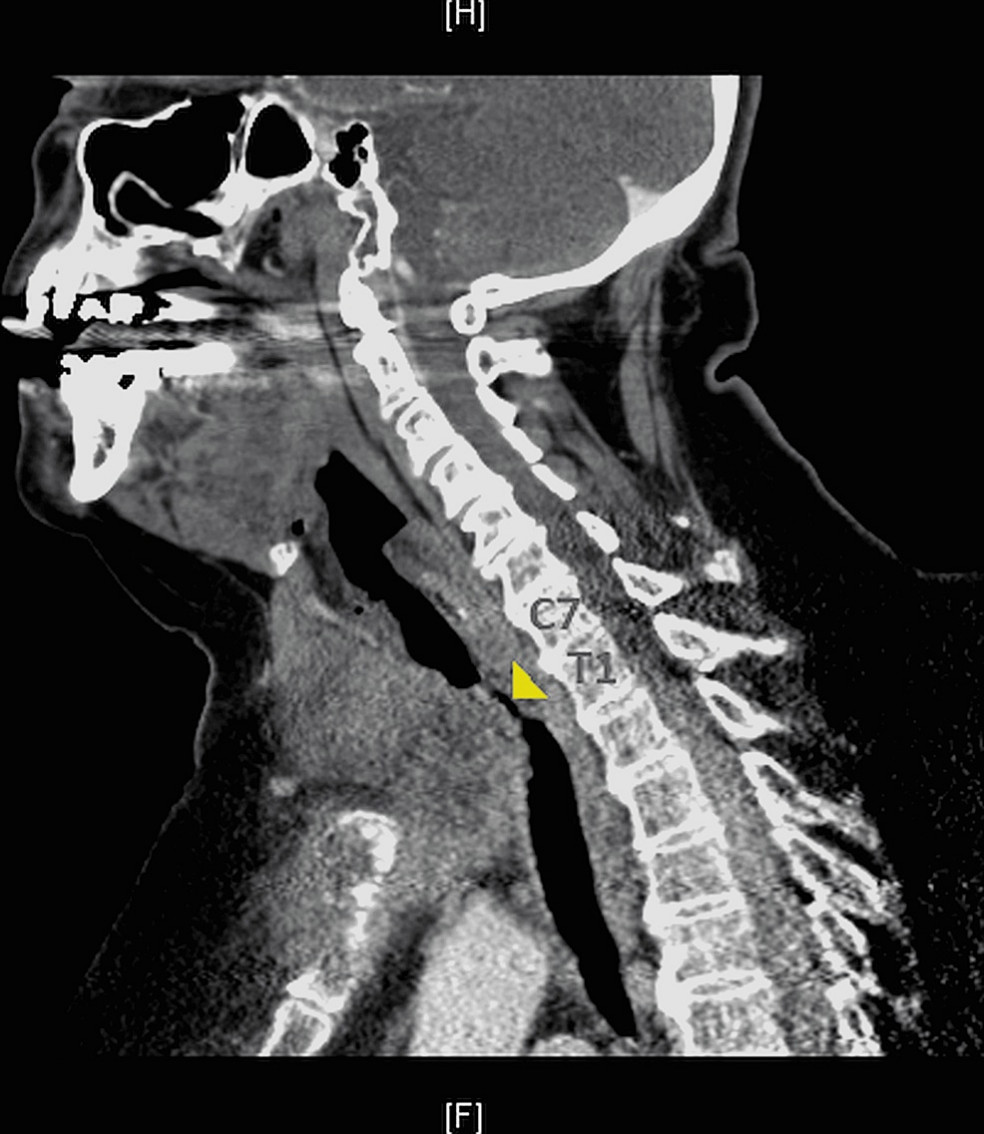
Sagittal CT image of the chest Tracheal stenosis (yellow arrow tip) is visible at the inferior C7 and T1 vertebral levels.

**Figure 2:**
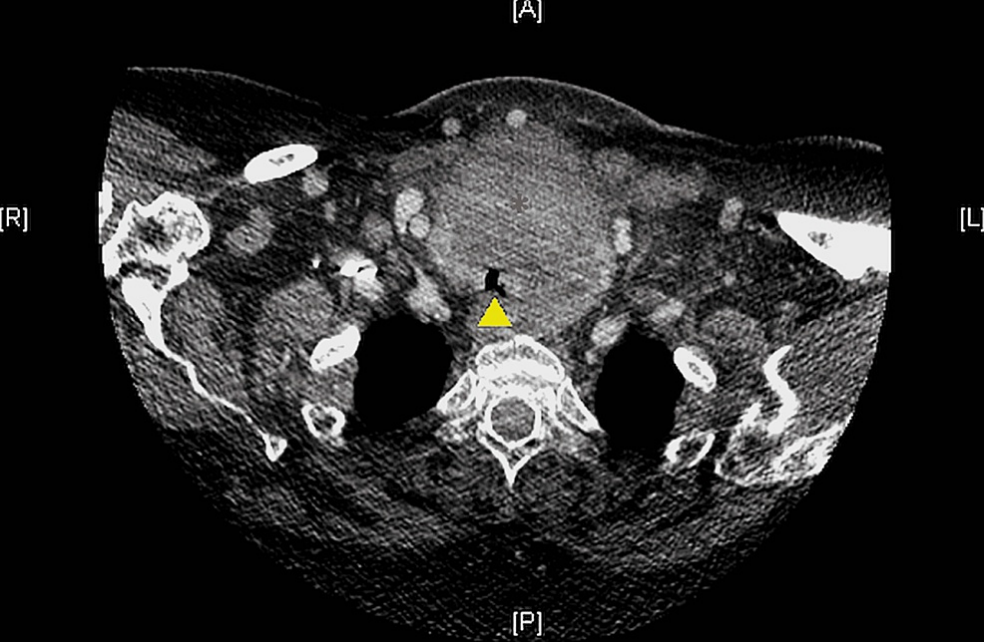
Coronal CT image of the chest at the T1 vertebral level Compression of the tracheal lumen (yellow arrow tip) by the mediastinal mass at its narrowest point can be seen.

A 70-year-old woman weighing 125 kilograms with a past medical history significant for untreated Graves’ disease, hypertension, paroxysmal atrial fibrillation, and morbid obesity (BMI 45) presented with a large mediastinal mass causing tracheal compression and respiratory distress. Her symptoms started six months ago and had since experienced worsening dyspnea when supine. She received intravenous steroid therapy at an outside hospital and was then transferred to the ICU at the authors’ institution. Computed tomography (CT) imaging showed a large soft tissue mass involving the anterior mediastinum and extending into the neck through the thyroid causing significant tracheal compression, sternal erosion, and multiple enlarged supraclavicular lymph nodes concerning for metastatic spread (Figure 1). At the C7-T1 level, the asymmetric tracheal compression was less than 4 millimeters horizontally but less than 9 millimeters vertically (Figure 2).

In the medical ICU, the patient was maintained in a seated position, and 10 liters/minute of heliox (70% helium, 30% oxygen) was initiated. There was an initial improvement in her respiratory status, but she still demonstrated audible stridor and laborious breathing. Due to the severity of the stenosis and expectant airway compromise, Anesthesiology, Interventional Pulmonology, Thoracic Surgery, and Otolaryngology Surgery consultations were urgently requested for airway management. Her short, thick neck, morbidly obese body habitus, large tongue, and positioning limitation suggested difficult airway placement. There was a concern for complete tracheal occlusion with the loss of spontaneous ventilation. In addition, the stenotic lumen may not accommodate the passage of our smallest available tracheal tube and fiberoptic bronchoscope. Since the tracheal compression had a distal location, a surgical airway would not bypass the critical stenosis. The location and size of the tumor prevented sternotomy or thoracotomy. Our major concern was causing airway bleeding from friable tissue in the trachea, and if fiberoptic intubation failed, the patient would rapidly go into respiratory failure. Therefore, the patient was brought to the operating room (OR) for planned veno-venous ECMO cannulation under monitored anesthetic care. Once access was obtained, we would administer intravenous medications for induction of general anesthesia and the patient would be intubated via rigid bronchoscopy. The patient verbally consented to our plan that included risks and benefits prior to transport from the ICU to the OR.

The patient was transferred over to the operating table, and standard ASA (American Society of Anesthesiologists) monitors were applied. Due to her severe dyspnea and inability to lie flat, we positioned her at a 45-degree angle at the hips to maintain adequate spontaneous ventilation. The patient was administered 100% oxygen via a non-rebreather mask, and an awake radial arterial line was placed. A liter of crystalloid and 250 milliliters of 5% albumin were administered for volume expansion in preparation for ECMO. Dexmedetomidine was administered at 8 microgram intervals for procedural sedation due to severe anxiety. The right superior vena cava (SVC) was accessed under ultrasound guidance by the surgeon, and the guidewire was threaded with difficulty due to her short, thick neck. Although the SVC was accessed successfully, femoral venous access was unable to be obtained. The guidewire could not be advanced despite multiple attempts in bilateral femoral veins or with repositioning to decrease the patient’s pelvic-femoral angle. This failure could have been due to the patient’s body habitus, poor access angle, or possible femoral vein thrombus. It was then suggested to place a bicaval dual-lumen catheter in the right SVC over the existing wire by the surgeon, but attempts were also unsuccessful secondary to the guidewire continuing to go into the right ventricle under fluoroscopic view. During these attempts, the patient was progressively requiring respiratory support. She was initially manually assisted with positive pressure ventilation while at a 45-degree head elevation, but she was poorly tolerating this maneuver due to anxiety. Later, the patient desaturated to a SpO2 of 76%, and therefore a size 5 laryngeal mask airway (LMA) was placed with inhalational sevoflurane for maintenance.

After failing two separate cannulation sites over an hour of operative time, the concern for increasing respiratory support and imminent airway collapse led to the multidisciplinary intraoperative decision to abort ECMO efforts and reassess our approach. A flexible fiberoptic intubation, while maintaining spontaneous ventilation in the sitting position, was pursued in hopes that the soft tissue mass would accommodate its passage. Rigid bronchoscopy was ready if the fiberoptic bronchoscope was unable to pass through the stenosis or if the patient acutely decompensated, but there was a high likelihood of trauma to the airway and a high chance of failure.

The LMA was removed and replaced with 100% inspired oxygen via facemask while the patient recovered from sevoflurane administration. A nasal approach was selected, and therefore a lubricated 30-French nasal airway that was cut along its length was inserted into the nare and a fiberoptic bronchoscope was passed through it. The vocal cords were anesthetized using 2% lidocaine (100 milligrams) through the bronchoscope port. The nasal airway was removed off of the fiberoptic bronchoscope. A lubed, 6-millimeter cuffed nasal Ring-Adair-Elwin tracheal tube, which has an outer diameter of 8.0 millimeters, was passed over the bronchoscope through the vocal cords and beyond the tracheal stenosis though with significant resistance. After successful placement and easy ventilation, midazolam 2 milligrams, propofol 200 milligrams, rocuronium 50 milligrams, and dexamethasone 8 milligrams were administered. After successful intubation, the patient was transported back to the ICU for further medical management. She was mechanically ventilated in the medical ICU for two days until successfully undergoing rigid bronchoscopy with tracheal stent placement for palliation. After the patient was extubated on hospital day 4, a decision was made not to undergo mass excision and the patient would explore radiation therapies. She was discharged on the seventh hospital day.

## Discussion

Though not typically considered a part of airway management, veno-venous ECMO has gained momentum in recent years as an option due to increased availability and the development of much safer, simpler ECMO machines [6]. Veno-venous ECMO can be used in patients with a tracheal mass, distal airway compression, or tracheal deformity, or when airway security cannot be obtained via traditional techniques [2-4]. In some scenarios, veno-venous ECMO is planned preemptively in anticipation of a difficult airway, allowing the ECMO procedure to be performed with advanced preparation and in a controlled environment [5]. Conversely, ECMO cannulation is also performed urgently or emergently following acute respiratory failure [6]. In both scenarios, veno-venous ECMO is frequently typified as a final recourse, and few, if any, anecdotal or standardized recommendations exist to guide the clinical approach to provide respiratory support should ECMO fail.

Veno-venous ECMO cannulation is dependent on successful establishment of central vascular access. An inability to gain vascular access and cannulate a patient requiring ECMO is a life-threatening complication [7]. Most cannulation-related complications are limited by experienced operators with ultrasound and fluoroscopy being utilized to increase success rates [8]. While the use and availability of ECMO in the United States has risen significantly over the past two decades, its availability for patients remains limited due to the extensive resources required to maintain an ECMO program [9]. As such, there are limited data surrounding some of the factors that influence cannulation failure. Expert opinion suggests that certain patient factors may prevent successful cannulation, including morbid obesity, low mean arterial pressures, hypovolemia, prior cannulation, vascular calcifications or scarring, and anatomical variants [7]. These factors may be similar to the factors that prevent successful central venous catheter placement, although there are limited prospective data to support predicting failure to cannulate for venous access based on patient characteristics [10].

The present case study represents one of the few published instances of a failed ECMO procedure wherein ECMO was intended to provide preemptive respiratory support in the setting of an uncertain airway. Despite this proactive strategy, planned respiratory support with veno-venous ECMO did not occur, and an alternative airway management strategy, with potentially significant complication risk, was urgently devised for this patient and situation. While this patient’s airway was ultimately secured without additional complications, this case study illustrates a contemporary need for evidence-based recommendations regarding airway management in patients with failed ECMO cannulation. As the availability of veno-venous ECMO increases, its role within the difficult airway algorithm and its limitations will need clarification. Since the inability of ECMO to cannulate may lead to a failure to rescue a patient with compromised perfusion or gas exchange, and thus morbidity or mortality, better guidance to help navigate such occurrences is needed.

## Conclusions

In summary, veno-venous ECMO is an effective approach to providing prolonged support to patients with respiratory failure. Its application in patients with significant airway obstruction is an infrequently reported yet potentially life-saving management strategy. However, when ECMO cannulation unexpectedly fails, a rapid multidisciplinary approach may be required to control the deteriorating airway. This case report illustrates both the potential role of ECMO within the airway management hierarchy and the persistent need for contingency planning should ECMO cannulation fail.
